# Clinical outcomes of endovascular therapy for aortoiliac artery chronic total occlusion via the transradial approach

**DOI:** 10.1186/s42155-025-00629-9

**Published:** 2025-12-13

**Authors:** Naoki Hayakawa, Toshiki Tsurumaki, Hiromi Miwa, Yasuyuki Tsuchida, Masanao Inoue, Shinya Ichihara, Satoshi Hirano, Shunsuke Maruta, Shunichi Kushida

**Affiliations:** 1 Department of Cardiovascular Medicine, Asahi General Hospital, I-1326, Asahi, Chiba 289-2511 Japan; 2 Department of Cardiology, Tsukuba Medical Center Foundation, Ibaraki, Japan

**Keywords:** Chronic total occlusion, Endovascular therapy, Transradial approach, Intravascular ultrasound, Aortoiliac artery

## Abstract

**Background:**

Endovascular therapy (EVT) for the aortoiliac (AI) artery using the transradial approach (TRA) has become increasingly common with the availability of radial-specific devices. However, the feasibility of treating AI chronic total occlusion (CTO) via the TRA remains unclear.

**Methods:**

This was a single-center, retrospective study. From October 2019 to November 2024, among 105 cases of AI CTO treated with EVT, 46 procedures performed via the TRA were analyzed. The primary endpoint was clinical success. The secondary endpoints were 12-month freedom from clinically driven target lesion revascularization (CD-TLR), successful antegrade guidewire passage, procedure time, need for femoral sheath insertion, and procedural or perioperative complications.

**Results:**

The mean age was 74.4 ± 9.2 years. Mean lesion length was 121.9 ± 44.1 mm, and 80.4% were classified as Trans-Atlantic Inter-Society Consensus II type C/D. The left radial approach was used in 91.3% of cases. Stent implantation was successful in all patients. Bare nitinol stents were used in 78.3% and covered stents in 21.7%. Intravascular ultrasound was used in 97.8% of procedures. The TRA alone was performed in 34.8%, the TRA with sheathless femoral access in 13.0%, and femoral sheath insertion in 52.2%. The 12-month rate of freedom from CD-TLR was 94.7%. Mean procedure time was 97.2 ± 52.3 min. Successful antegrade guidewire passage was achieved in 56.5%. Procedural and perioperative complications each occurred in 6.6%. No cases of radial artery occlusion, cerebral infarction, or blue toe syndrome were observed. In the multivariable analysis, common-to-external iliac artery CTO (adjusted odds ratio 0.09, 95% confidence interval 0.02–0.53, *p* = 0.008) and common femoral artery involvement (adjusted odds ratio 0.05, 95% confidence interval 0.006–0.39, *p* = 0.005) were independently associated with unsuccessful antegrade guidewire passage.

**Conclusion:**

EVT for AI CTO via the TRA is feasible and achieves high procedural success; however, many cases required an additional bidirectional approach using the transfemoral route.

## Background

Endovascular therapy (EVT) for the aortoiliac (AI) region plays a pivotal role in the treatment of lower extremity artery disease and has become the first-line therapy for AI occlusive disease [1, 2]. Traditionally, the transfemoral approach (TFA) has been the standard access site for EVT; however, puncture-related complications and the need for prolonged bed rest remain significant concerns [3]. By contrast, the transradial approach (TRA)—well established in coronary interventions for its safety and patient comfort—has recently been adopted for peripheral vascular interventions [4, 5]. The TRA is associated with fewer bleeding complications, earlier ambulation, and improved patient satisfaction. Recent studies have shown that in the AI region, the TRA offers perioperative safety comparable to the TFA, while providing the added benefits of easier hemostasis and earlier mobilization [6, 7]. Moreover, the TRA has been reported to result in shorter procedural times than the TFA [7]. Regarding long-term durability, Tsuchida et al. [8] recently reported favorable 3-year outcomes using the TRA-specific bare-nitinol stent Misago for AI lesions. Similarly, a multicenter study from Japan demonstrated that TRA stenting for iliac artery lesions was as safe and feasible as TFA, underscoring the clinical applicability of TRA in this vascular territory [9].

Nevertheless, several technical challenges persist in EVT for iliac arteries, particularly in complex chronic total occlusion (CTO) lesions [10]. These include insufficient device support, limited guidewire (GW) maneuverability, and difficulty delivering large-caliber devices. To address these limitations, a recent report demonstrated the safety and feasibility of a bidirectional strategy combining the TRA with sheathless femoral access, thereby compensating for the device support and reach limitations of the TRA [11, 12].

Despite these advances, evidence specifically focused on iliac CTO remains limited. In particular, which CTO cases can be successfully treated with the TRA alone and which require a bidirectional approach has not been fully clarified. Therefore, the aim of the present study was to evaluate the clinical outcomes of EVT for AI artery CTO performed via TRA and to clarify its feasibility.

## Methods

### Study population and design

**Fig. 1:**
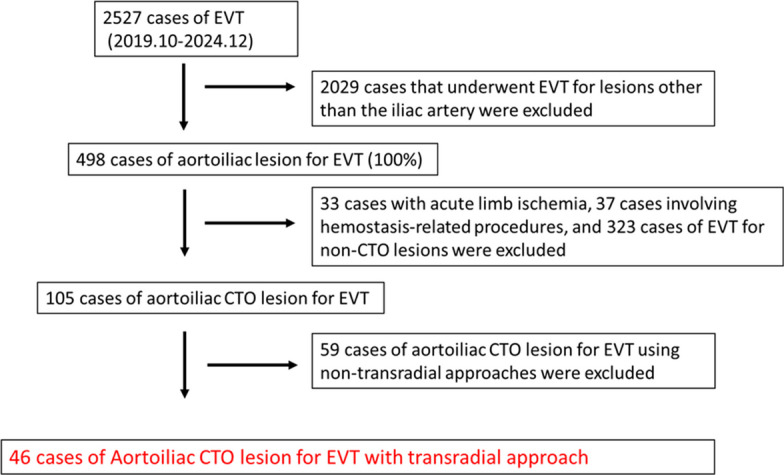
Study flow chart

This single-center, retrospective study was conducted at Asahi General Hospital. Between November 2019 and December 2024, a total of 2527 patients underwent EVT at our institution. After excluding 2029 patients treated for lesions outside the iliac artery, 33 patients with acute limb ischemia, 37 patients who underwent hemostasis-related procedures, and 323 patients with non-CTO lesions, 105 patients with AI CTO were identified for analysis. Of these, 59 patients treated with non-transradial approaches were excluded, leaving 46 patients with AI CTO treated via the TRA included in the final analysis (Fig. 1).

The choice of TRA was determined at the discretion of each operator. Patients were excluded if the TRA was considered infeasible during pre-procedural assessment, defined as the absence of a palpable radial pulse, a radial artery (RA) diameter of less than 2 mm on ultrasound, or unsuitability as judged by the operator.

The study protocol was approved by the local ethics committee of Asahi General Hospital and conducted in accordance with the Declaration of Helsinki. The requirement for informed consent was waived because of the retrospective design using existing medical records; however, patients were given the opportunity to opt out. Relevant study information was made publicly available in accordance with the Ethical Guidelines for Medical and Health Research Involving Human Subjects.

### Procedural protocol

Two antithrombotic agents were administered at least 24 h before the procedure. Aspirin, clopidogrel, or prasugrel was primarily used. In cases of allergy or previous bleeding events associated with these drugs, cilostazol was used as an alternative antiplatelet agent. Anticoagulants were prescribed when indicated, such as in patients with atrial fibrillation or other clinical needs.

EVT was performed for AI CTO lesions. The primary access site was the RA, selected according to the pre-procedural feasibility assessment described above. When lesion crossing or device delivery was judged difficult with TRA alone, femoral puncture was performed to establish a bidirectional approach. The decision to insert a sheath or microcatheter from the femoral access, as well as the choice of sheath size, was made at the operator’s discretion based on lesion characteristics and procedural requirements.

After sheath insertion into the RA and/or common femoral artery (CFA), 5000 IU of unfractionated heparin was administered. When procedures were performed via the TRA, a Slender sheath with guiding catheter (Slenguide; Terumo Corp. Tokyo, Japan), a Destination Slender guiding sheath (Terumo Corp.), or a Parent 45 guiding sheath (Medikit Corp.) was used. Lesion crossing was attempted using a 0.014-, 0.018-, or 0.035-inch GW with a microcatheter or back-up support catheter. Several types of 0.014-inch GWs, including Gladius (Asahi Intec. Aich, Japan), Gladius MGES (Asahi Intec.), Jupiter FC (Boston Scientific. Marlborough, MA, USA), Jupiter S6 (Boston Scientific.), Jupiter X (Boston Scientiffic.), Halberd (Asahi Intec.), Astato XS9-40 (Asahi Intec.), Crosslead penetration (Asahi Intec.), and Jupiter T45 (Boston Scientific.), were used. Several types of 0.018-inch GWs, including Gladius MG18 (Asahi Intec.), Astato 18 (Asahi Intec.), and Crosslead 18 (Asahi Intec.), were used. A 0.035-inch GW, either Radifocus (Terumo Corp.) or Crosslead 35(Asahi Intec.), was used.

A bidirectional approach was adopted if conventional antegrade GW crossing failed. After successful GW passage, the balloon size for pre- and post-dilatation, as well as the type and diameter of stents, were determined based on quantitative vascular angiography and/or intravascular ultrasound (IVUS) evaluation. Stents were implanted from a healthy-to-healthy segment to ensure full coverage of the atherosclerotic lesion. Bare-nitinol stents (BNS), including Misago (Terumo Corp.), SMART (Cardinal Health Inc., Dublin, OH, USA), EPIC (Boston Scientific.), E-Luminex (Becton, Dickinson and Company, Franklin Lakes, NJ, USA), and Everflex (Medtronic Inc., Plymouth, MN, USA), were used. Covered stents (CS), such as Viabahn VBX (WL Gore & Associates, Bloomington, IL, USA) and Lifestream (Becton, Dickinson and Company), were also utilized. When measured by IVUS, a BNS with a diameter approximately equivalent to the mean external elastic membrane size of the target vessel, or a CS matching the luminal size, was selected. The size of the pre-dilation balloon was chosen to be smaller than the luminal diameter of the reference segment of the target vessel, while the size of the post-dilation balloon was selected to match the diameter of the implanted stent.

Device selection—including the guiding sheath, GW, balloon catheter, and stent type—was left to the discretion of the treating operator.

Procedural success was defined as successful recanalization of the target lesion with < 30% residual stenosis on final angiography. Hemostasis at the access site was achieved using either manual compression or a closure device, according to access route and operator preference.

### Study endpoints and definitions

The primary endpoint of this study was clinical success, defined as < 30% residual stenosis without angiographic flow limitation. The secondary endpoints were the 12-month rate of freedom from clinically driven target lesion revascularization (CD-TLR), successful antegrade GW passage, procedure time, need for femoral sheath insertion, and procedural or perioperative complications. Procedure time was defined as the interval from the administration of local anesthesia for the initial puncture to the complete removal of the sheath at the end of the procedure. Shaggy aorta was defined as a diffuse, irregularly shaped atherosclerotic change involving more than 75% of the length of the aorta from the aortic arch to the visceral segment with atherosclerotic plaque thickness greater than 4 mm, as confirmed by contrast enhanced CT [13]. All procedures and clinical event assessments were conducted at our institution by at least three specialists certified by the Japanese Association of Cardiovascular Intervention and Therapeutics.

### Statistical analysis

Statistical analyses were performed using JMP software version 13.0 (SAS Institute, Cary, NC, USA). Continuous variables are presented as mean ± standard deviation, and categorical variables are presented as count and percentage. A *p* value of < 0.05 was considered statistically significant, and 95% confidence intervals (CIs) are reported where appropriate. Differences in baseline characteristics among groups were tested using one-way analysis of variance for continuous variabilities and the chi-squared test for categorical variables. The rate of freedom from CD-TLR was estimated using the Kaplan–Meier method. The association between baseline characteristics and failure of antegrade GW passage was evaluated using a Cox proportional hazards regression model. Variables showing statistical significance in the univariable analysis were entered into the subsequent multivariable model.

## Results

### Baseline characteristics

The patients’ clinical characteristics are summarized in Table 1. In total, 46 patients who underwent EVT for AI CTO via the TRA were included in the analysis. Their mean age was 74.4 ± 9.2 years, and 39 patients (84.8%) were male. Twelve patients (26.1%) presented with chronic limb-threatening ischemia. The mean patient height was 161.2 ± 8.4 cm, and the mean body weight was 53.6 ± 11.4 kg. Ambulatory status was preserved in 91.3% of patients. Regarding comorbidities, hypertension was present in 78.3%, diabetes mellitus in 32.6%, and hemodialysis in 4.4% of patients. The mean number of treated lesions per patient was 2.2 ± 1.2. The most frequent lesion distribution was common iliac artery (CIA)–external iliac artery (EIA) (39.1%), followed by CIA alone (21.7%) and CIA–CFA (10.9%). The mean lesion length was 121.9 ± 44.1 mm. According to the Trans-Atlantic Inter-Society Consensus (TASC) II classification, 37 lesions (80.4%) were categorized as type C or D, indicating high lesion complexity. Preprocedural enhanced computed tomography (CT) was performed in 87.0% of patients, and a shaggy aorta was identified in 4.3%. A proximal blunt cap was observed in 56.5% of cases, while proximal calcification—defined as bilateral calcification at the proximal cap of the CTO, identified either on preprocedural CT or intra-procedural angiography—was present in 26.1%.

**Table 1:** Patient and lesion characteristics

Patient and lesion characteristics	*n* = 46
Age, years	74.4 ± 9.2
Male	39 (84.8)
CLTI	12 (26.1)
Height, cm	161.2 ± 8.4
Weight, kg	53.6 ± 11.4
Ambulatory	42 (91.3)
CAD	18 (39.1)
CVD	17 (38.0)
HT	36 (78.3)
DL	26 (56.5)
DM	15 (32.6)
CKD	9 (19.6)
HD	2 (4.4)
Smoking	39 (84.8)
Rutherford classification	
3	31 (67.4)
4	3 (6.5)
5	11 (23.9)
6	1 (2.2)
Number of treated lesions	2.2 ± 1.2
Aorta limited	0 (0.0)
Aorta–CIA	2 (4.3)
Aorta–EIA	2 (4.3)
CIA	10 (21.7)
CIA–EIA	18 (39.1)
CIA–CFA	5 (10.9)
EIA	6 (13.0)
EIA–CFA	2 (4.3)
Lesion length	121.9 ± 44.1
TASC II	
A	0 (0.0)
B	9 (19.6)
C	8 (17.4)
D	29 (63.0)
C/D	37 (80.4)
Preprocedural enhanced CT	40 (87.0)
Shaggy aorta	2 (4.3)
Proximal cap blunt	26 (56.5)
Proximal calcification	12 (26.1)

Data are presented as mean ± standard deviation or *n* (%)

*CLTI* chronic limb-threatening ischemia, *CAD* coronary artery disease, *CVD* cerebrovascular disease, *HT* hypertension, *DM* diabetes mellitus, *DL* dyslipidemia, *CKD* chronic kidney disease, *HD* hemodialysis, *CIA* common iliac artery, *EIA* external iliac artery, *CFA* common femoral artery, *TASC* Trans-Atlantic Inter-Society Consensus, *CT* computed tomography

### Outcome measures

The procedural outcomes are summarized in Table 2. The left RA was the predominant access site, used in 91.3% of cases, while the right RA and distal RA were each used in 8.7%. The mean guiding sheath size was 6.0 ± 0.2 Fr. Femoral sheath insertion was performed in 52.2% of patients, and a bidirectional approach was required in 65.2%. In 13.0% of cases, a sheathless femoral technique was employed. IVUS was used in nearly all procedures (97.8%).

**Table 2:** Procedure characteristics

Lt RA	42 (91.3)
Rt RA	4 (8.7)
DRA	4 (8.7)
Guiding sheath Fr	6.0 ± 0.2
Destination SL	36 (78.3)
Slenguide	9 (19.6)
Parent 45	1 (2.2)
Femoral sheath insertion	24 (52.2)
Bi-directional	30 (65.2)
Sheathless femoral technique	6 (13.0)
Support or diagnostic catheter use	26 (56.5)
Micro catheter use	44 (95.7)
IVUS use	45 (97.8)
Subintimal involvement	2 (4.3)
CTf3D-RM	29 (63.0)
Antegrade wiring success	26 (56.5)
Guidewire tip load, g	16.3 ± 19.4
Guidewires, *n*	2.7 ± 1.6
Guidewire crossing time, min	38.0 ± 42.1
Procedure time, min	97.2 ± 52.3
Stent use	46 (100)
Stents, *n*	2.3 ± 1.2
Stent diameter, mm	9.0 ± 0.7
Stent length, mm	136.3 ± 48.8
Final device	
Misago	27 (58.7)
SMART	3 (6.5)
BNS only	36 (78.3)
CS use	10 (21.7)
CFA DCB use	10 (21.7)

Data are presented as *n* (%) or mean ± standard deviation

*Lt* left, *Rt* right, *RA* radial artery, *DRA* distal radial artery, *SL* Slender, *IVUS* intravascular ultrasound, *CTf3D-RM* computed tomography fusion three-dimensional roadmap, *BNS* bare nitinol stent, *CS* covered stent, *CFA* common femoral artery, *DCB* drug-coated balloon

Antegrade GW passage was successful in 56.5% of cases. The mean GW tip load was 16.3 ± 19.4 g, and the mean number of GWs used per case was 2.7 ± 1.6. The mean GW crossing time was 38.0 ± 42.1 min, while the mean total procedure time was 97.2 ± 52.3 min.

Stents were implanted in all patients (100%). The mean number of stents per patient was 2.3 ± 1.2, with a mean diameter of 9.0 ± 0.7 mm and a mean total stent length of 136.3 ± 48.8 mm. The Misago stent (Terumo Corp.) was the most frequently used device, implanted in 58.7% of cases. A balloon-expandable covered stent was used in 21.7%, and drug-coated balloon angioplasty for the CFA was performed in another 21.7%.

The clinical outcomes are summarized in Table 3. Clinical success was achieved in all 46 patients (100%). Procedural complications occurred in 6.6% of cases, including two cases of vessel perforation (4.3%) and one case of distal embolism (2.2%). The details of the perforation cases are as follows. In the first patient, the GW traversed the subintimal space in a heavily calcified CTO lesion. Although a CS was implanted, bleeding occurred from the stent edge, which was successfully managed by the additional placement of another CS as a bailout. In the second patient, despite the absence of severe calcification, the GW advanced along the vessel margin. After deployment of a BNS, post-dilation induced oozing, likely due to overexpansion in an eccentrically dilated stent. The bleeding was controlled by implanting a CS within the BNS as a bailout measure. All three were successfully managed with intra-procedural bailout measures, and none resulted in major postoperative adverse events. No puncture site complications, cerebral infarction, blue toe syndrome, or RA occlusion (RAO) were observed (all 0%). Periprocedural complications occurred in three patients (6.6%), while non-procedural adverse events included urinary tract infection (2.2%), cerebral hemorrhage (2.2%), and death due to congestive heart failure (2.2%).

**Table 3:** Clinical outcomes

Clinical success	46 (100)
Procedural complication	3 (6.6)
Vessel perforation	2 (4.3)
Distal embolism	1 (2.2)
Periprocedural complication	3 (6.6)
Puncture site complication	0 (0.0)
Cerebral infarction	0 (0.0)
Blue toe syndrome	0 (0.0)
Radial artery occlusion	0 (0.0)
UTI	1 (2.2)
Cerebral hemorrhage	1 (2.2)
Death due to CHF	1 (2.2)
Time of bed rest, min	218.5 ± 155
Pre-ABI	0.52 ± 0.18
Post-ABI	0.99 ± 0.12
Follow-up, days	576.5 (342.3–1031)

Data are presented as *n* (%), mean ± standard deviation, or median (range)

*UTI* urinary tract infection, *CHF* congestive heart failure, *ABI* ankle–brachial index

The mean bed rest time was 218.5 ± 155 min (less than 4 h in most cases). The ankle-brachial index significantly improved from 0.52 ± 0.18 before EVT to 0.99 ± 0.12 after the procedure.

**Fig. 2:**
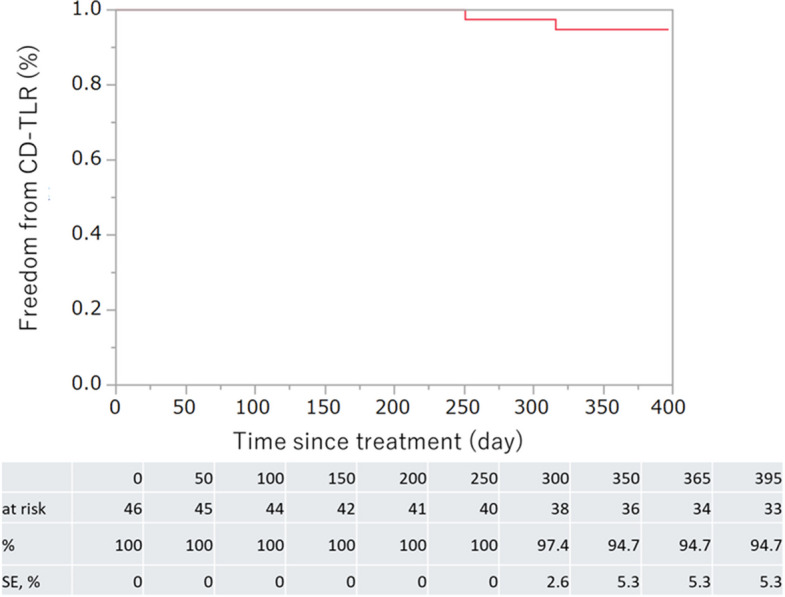
Kaplan–Meier curves of freedom from clinically driven target lesion revascularization

At 12 months, the Kaplan–Meier estimate demonstrated a rate of freedom from CD-TLR of 96.9% (Fig. 2).

Table 4 summarizes the risk factors for unsuccessful antegrade GW passage. In the univariable analysis, CIA–EIA occlusion (odds ratio [OR] 0.30, 95% CI 0.09–1.04, *p* = 0.07), lesion length of ≥ 125 mm (OR 0.21, 95% CI 0.06–0.75, *p* = 0.02), TASC II type C/D (OR 0.12, 95% CI 0.01–1.04, *p* = 0.06), and CFA involvement (OR 0.24, 95% CI 0.05–1.10, *p* = 0.08) were identified as potential predictors of failure in antegrade GW passage. In the multivariable analysis, CIA–EIA CTO (adjusted OR 0.09, 95% CI 0.02–0.53, *p* = 0.008) and CFA involvement (adjusted OR 0.05, 95% CI 0.006–0.39, *p* = 0.005) emerged as independent predictors of unsuccessful antegrade GW passage. These findings suggest that long CTO and CFA involvement are key determinants of the need for a bidirectional strategy.

**Table 4:** Predictors of antegrade guidewire passage

	**Univariable analysis**		**Multivariable analysis**	
	**Odds ratio (95% CI)**	***p* value**	**Odds ratio (95% CI)**	***p* value**
CFA involvement	0.24 (0.05–1.10)	0.08	0.05 (0.006–0.39)	0.005
CIA	4.0 (0.74–21.49)	0.15		
CIA–EIA	0.30 (0.09–1.04)	0.07	0.09 (0.02–0.53)	0.008
CIA–CFA	0.16 (0.02–1.56)	0.15		
EIA	4.52 (0.48–42.27)	0.21		
CTf-3DRM	0.39 (0.11–1.39)	0.22		
Lesion length ≥ 125 mm	0.21 (0.06–0.75)	0.02		
TASC II C/D	0.12 (0.01–1.04)	0.06		
Blunt type occlusion	1.12 (0.34–3.61)	1.00		
Proximal calcification	0.44 (0.16–1.69)	0.31	0.19 (0.03–1.12)	0.07
Preprocedural CT	3.0 (0.49–18.36)	0.38		
Ambulatory	4.41 (0.42–46.65)	0.30		
CAD	0.94 (0.28–3.09)	1.00		
CVD	0.79 (0.24–2.65)	0.76		
HT	0.83 (0.20–3.47)	1.00		
DM	0.55 (0.16–1.92)	0.53		
DL	0.54 (0.16–1.78)	0.38		
CKD	1.70 (0.37–7.85)	0.71		
HD	0.76 (0.04–12.95)	1.00		
Smoking	0.18 (0.02–1.60)	0.12		

*CI* confidence interval, *CFA* common femoral artery, *CIA* common iliac artery, *EIA* external iliac artery, *CTf-3DRM* computed tomography fusion three-dimensional roadmap, *TASC* Trans-Atlantic Inter-Society Consensus, *CT* computed tomography, *CAD* coronary artery disease, *CVD* cerebrovascular disease, *HT* hypertension, *DM* diabetes mellitus, *DL* dyslipidemia, *CKD* chronic kidney disease, *HD* hemodialysis

## Discussion

In this single-center study, we demonstrated that the TRA for EVT of AI CTO is both feasible and effective, achieving a high rate of clinical success with a low incidence of access site–related complications. Previous studies of the TRA for AI lesions have primarily focused on simpler cases, leaving its role in complex lesions such as CTO insufficiently defined [6, 9, 10, 14–16]. The present findings suggest that EVT via the TRA is feasible when appropriate patient selection and technical adjustments are made. To our knowledge, no prior clinical study has focused exclusively on AI CTO treated via the TRA while also examining both procedural feasibility and predictors of antegrade GW passage failure.

The TRA offers several advantages, including reduced access-site bleeding complications, earlier ambulation, and improved patient comfort [5–7]. Therefore, in lesions where comparable clinical outcomes can be achieved, the less invasive TRA may be considered a favorable option. On the other hand, the TFA remains preferable in cases with unfavorable aortic arch anatomy, small RA diameter, or when a larger guiding system is required, such as during the placement of large-diameter CS.

Careful preprocedural assessment is essential for the safe and effective application of the TRA in CTO treatment. Cases that are generally considered challenging for transradial EVT include patients on hemodialysis and those with a type III aortic arch, a shaggy aorta, or marked aortic tortuosity. The reverse wire technique for type III arch anatomy and the distal RA approach in patients undergoing hemodialysis are reported strategies to overcome these difficulties; however, in the present study, the vast majority of procedures were performed via the RA, and only 4.4% of patients were on hemodialysis; therefore, the need for such techniques was limited [17, 18]. In any case, careful preprocedural access evaluation—including CT imaging—remains crucial when planning the TRA for CTO interventions. Prior to performing TRA for AI CTO, it is crucial to understand the anatomy of the aortic arch and the anatomical variations of the RA. Accordingly, preprocedural evaluation using ultrasound, magnetic resonance angiography (MRA), or CT is recommended [19].

Furthermore, the routine use of preprocedural CT imaging (87.0%) allowed detailed evaluation of proximal cap morphology, calcification, and overall lesion anatomy, which may have contributed to improved procedural efficiency and more informed access planning [20]. In cases approached via the RA, CT imaging also likely supported risk stratification for antegrade access and prediction of procedural difficulty. When anatomical factors such as aortic tortuosity suggested that an antegrade-only strategy would be challenging, femoral sheath insertion was likely considered from the outset.

Importantly, our analysis identified specific lesion characteristics—namely CIA–EIA involvement and CFA inclusion—as independent predictors of unsuccessful antegrade GW passage. In the univariate analysis, lesion length ≥ 125 mm was significantly associated with difficulty in antegrade GW passage; however, this association was not observed in the multivariate analysis. In this study, lesion length did not necessarily correspond to the total occlusion length, and it is possible that some cases with long lesions but relatively short occlusion lengths were included. On the other hand, CIA–EIA occlusion clearly indicates a long occlusive segment, which may explain the result observed in the multivariate analysis. Nevertheless, because of the limited sample size, it was difficult to include multiple variables in the multivariate model. Further studies with a larger number of cases may enable a more detailed evaluation of lesion and occlusion lengths. In practice, a bidirectional approach was employed in cases where the antegrade GW failed to cross the occlusion due to severe calcification or vessel tortuosity, or when re-entry into the true lumen could not be achieved from the radial side, even with the use of IVUS. In such situations, a retrograde approach via the femoral artery enabled successful guidewire passage and lesion crossing. These findings suggest that while the TRA can be successfully applied in a considerable proportion of complex iliac lesions, patients with long CTO, combined CIA–EIA occlusions, or CFA involvement may benefit from an upfront bidirectional strategy rather than attempting the TRA alone. A combined TRA and retrograde approach has been reported as an effective option for complex iliac lesions, with the retrograde route established either with or without a sheath [12].

Although IVUS was used in nearly all cases in this study, its efficacy in AI stenting has not always been clearly demonstrated in previous research [21]. However, those earlier studies included a broad spectrum of AI lesions—many of which were stenotic rather than totally occluded—and did not specifically address CTO. In the context of CTO, where GW crossing and accurate evaluation of lesion morphology, calcification, and vessel diameter are particularly challenging, the clinical value of IVUS may be especially significant. Indeed, prior studies have demonstrated the benefits of IVUS-guided EVT for complex AI occlusions [22]. In our cohort, despite the high lesion complexity—with more than 80% classified as TASC II type C/D—the high procedural success rate and relatively short procedure time may, at least in part, be attributable to the consistent use of IVUS.

Bed rest time was remarkably short (< 4 h in most patients), underscoring one of the major clinical advantages of the TRA compared with transfemoral access. Although more than half of the cases required a bidirectional approach, the relatively short bed rest duration may be explained by the fact that nearly 10% of patients underwent a sheathless femoral technique [12]. Even when a femoral sheath was used, hemostasis was often achieved intraoperatively using closure devices, in combination with balloon inflation from the TRA to temporarily occlude blood flow and facilitate hemostasis at the femoral puncture site.

Although 65.2% of patients required a bidirectional approach, this strategy was not uniformly employed as a last resort following exhaustive antegrade wiring attempts. Rather, the decision to introduce femoral access was made at the operator’s discretion and often at an appropriate timing—before prolonged or excessive antegrade attempts. This likely contributed to the observation that both GW crossing time (38.0 ± 42.1 min) and total procedural time (97.2 ± 52.3 min) remained within acceptable limits despite the high lesion complexity. These findings suggest that timely transition to a bidirectional strategy was effective in maintaining both procedural efficiency and safety.

Conversely, it is possible that if operators had persisted longer with the antegrade-only strategy, a greater number of cases might have achieved technical success without additional access. However, such an approach would likely have increased procedure time and the risk of complications. Thus, our results emphasize the importance of balancing persistence with antegrade strategies against the timely adoption of a bidirectional approach—tailored to lesion morphology, procedural progress, and overall patient safety.

In the present study, the high procedural success rate was accompanied by a favorable rate of 1-year freedom from CD-TLR. Although the Misago stent was predominantly used, our findings align with the report by Tsuchida et al. [8], which demonstrated favorable outcomes of Misago stent implantation via the TRA in the AI segment. In addition, covered stents were employed in 21.7% of cases. Previous studies have reported excellent results with balloon-expandable covered stents in complex lesions [23–25]. With the growing availability of these devices for use via the TRA in recent years, EVT can now be performed even in more challenging situations, such as heavily calcified disease or long contiguous occlusions extending from the aorta—factors that may partly explain the favorable outcomes observed in this cohort.

In this study, both intraprocedural and periprocedural complications were relatively infrequent. All intraprocedural complications were successfully managed with bailout strategies and did not adversely affect postoperative outcomes. For major complications such as vessel perforation or distal embolization, the expanded availability of covered stents via the TRA has increased therapeutic flexibility; nonetheless, it remains essential to employ the TFA without hesitation when complications arise. Vessel perforation is one of the most serious complications in AI EVT, and its reported incidence in iliac artery interventions ranges from 0.8 to 3.0% [5, 25, 26]. In the present study, two cases of perforation were observed. One occurred after stent deployment following subintimal wiring in a severely calcified occlusion, while the other occurred after post-dilatation of an eccentrically placed BNS. Neither of these cases represented findings unique to the TRA, as such events could readily occur even with TFA. Reported risk factors for vessel perforation include calcification, CTO lesions, excessive balloon dilatation, female sex, and steroid use [26]. From a pathophysiological perspective in CTO lesions, over-dilatation of a chronically narrowed occluded segment or aggressive expansion after guidewire passage through the vessel margin or subintimal space likely increases the risk of perforation as was seen in both of the present cases. To prevent such events, it is important to precisely measure vessel diameter using CT or IVUS to avoid excessive balloon inflation. In addition, the prophylactic or bailout use of covered stents can serve as an effective countermeasure when high-risk conditions are anticipated. In the event of an iliac artery rupture, our management strategy is as follows. Even when the procedure is performed via the TRA, we always prepare the inguinal region in advance with sterile draping to allow for immediate femoral access if necessary. Once bleeding is recognized, we first perform rapid balloon occlusion of the rupture site using an appropriately sized balloon via the TRA system to promptly stop blood flow. While the upstream flow is temporarily blocked, an 8Fr or larger sheath is inserted through the femoral artery in preparation for the delivery of a large-caliber CS. Since most CS require sheaths of 8Fr or larger, delivery through a standard 6Fr radial system is generally difficult. We do not use 8Fr radial sheaths because of the increased risk of RA injury. Recently, 6Fr-compatible CS have become available, and depending on the vessel diameter and the extent of bleeding, it may be possible to deliver a CS via the 6Fr TRA system. In such cases, it is still crucial to achieve balloon occlusion of the rupture site first; therefore, a situation may arise in which temporary balloon occlusion is performed via the TFA while a 6Fr-compatible CS is deployed from the TRA. In any case, while the TRA is a minimally invasive access route, it is essential to ensure that rapid femoral access can be obtained immediately in preparation for potential vessel rupture.

Furthermore, no cases of RAO, periprocedural stroke, or access-site bleeding complications were observed—results that are even more favorable than those reported previously. Although the TRA offers several procedural advantages, RAO remains one of its major limitations. The reported incidence of RAO after TRA ranges from 1 to 12%, and previous studies have identified several risk factors and proposed strategies to reduce its occurrence [27]. One of the intrinsic limitations of the TRA is its dependency on the relatively small caliber of the RA. Use of a sheath exceeding the inner luminal diameter of the RA has been identified as a potential risk factor for vessel injury and subsequent RAO [28]. Furthermore, previous reports have indicated that the lack of patent hemostasis, inadequate heparin dosing, prolonged hemostasis duration, and procedural pain contribute to the risk of RAO [29, 30]. Although no cases of RAO were observed in the present study, this favorable outcome may be partly attributable to the fact that, during pre-procedural evaluation for EVT, vessels with a radial artery diameter of ≥ 2.0 mm were selected in most cases, and gradual decompression was performed during sustained hemostasis. However, the number of cases was small, and this was a retrospective analysis conducted at multiple centers without a standardized protocol. Therefore, further investigation is required to identify the factors that can help prevent the occurrence of RAO during TRA-EVT. To prevent RAO during TRA-EVT, it is important to evaluate the RA size preoperatively using ultrasound and to select an appropriately sized guiding sheath accordingly. During the procedure, adequate administration of heparin should be ensured to prevent thrombotic occlusion. The use of vasodilators and analgesics as needed can help with pain control. In addition, adopting hemostasis techniques that avoid excessive compression of the arterial lumen may help reduce the risk of RAO.

Although the small sample size is a limitation, these findings may partly reflect careful pre-procedural access evaluation and the predominant use of the left radial approach [5, 6, 9].

## Limitations

This study has several limitations. First, it was a single-center, retrospective, nonrandomized analysis with a relatively small sample size, which limits the generalizability of the results. Second, the choice of access strategy, timing of conversion to a bidirectional approach, and device selection (including the type of stent or covered stent) were left to the operator’s discretion, without a standardized protocol. Third, patient selection was based on operator judgment of RA feasibility, which may have introduced selection bias. Fourth, all angiographic findings, IVUS evaluations, and clinical outcomes were assessed on-site, without adjudication by an independent core laboratory or clinical events committee. Fifth, although IVUS was used in nearly all procedures, its interpretation and procedural contribution were not systematically analyzed, so its specific impact cannot be definitively determined. Finally, the relatively short follow-up period limits the ability to evaluate long-term patency and clinical durability beyond 12 months. Future prospective, multicenter studies with larger cohorts, standardized procedural protocols, and independent outcome adjudication are needed to validate these findings and further define the role of the TRA and bidirectional strategies in complex AI CTO interventions.

## Conclusions

Our study has demonstrated that EVT for AI CTO using the TRA is feasible and achieves a high procedural success rate. Nevertheless, a substantial proportion of cases required the addition of a bidirectional approach with the TFA. Lesion characteristics—particularly long CTO (> 125 mm) and CIA–EIA occlusion—were associated with antegrade failure, suggesting that a planned bidirectional strategy should be considered in such situations. Taken together, these findings support the concept that the TRA may serve as a first-line access option in selected patients with AI CTO, provided that lesion complexity and access planning are carefully assessed.

## Data Availability

The datasets used and/or analyzed during the current study are available from the corresponding author upon reasonable request.

## Abbreviations

EVT: Endovascular therapy
AI: Aortoiliac
TRA: Transradial approach
CTO: Chronic total occlusion
CD-TLR: Clinically driven target lesion revascularization
TFA: Transfemoral approach
GW: Guidewire
RA: Radial artery
CFA: Common femoral artery
IVUS: Intravascular ultrasound
Cis: Confidence intervals
CIA: Common iliac artery
EIA: External iliac artery
TASC: Trans-Atlantic Inter-Society Consensus
CT: Computed tomography
